# Enterolactone has stronger effects than enterodiol on ovarian cancer

**DOI:** 10.1186/s13048-017-0346-z

**Published:** 2017-07-24

**Authors:** Huidi Liu, Jianrui Liu, Siwen Wang, Zheng Zeng, Ting Li, Yongfang Liu, Emilio Mastriani, Qing-Hai Li, Hong-Xia Bao, Yu-Jie Zhou, Xiaoyu Wang, Sijing Hu, Shan Gao, Yingying Qi, Zhihang Shen, Hongyue Wang, Miao Yu, Tingting Gao, Randal N. Johnston, Shu-Lin Liu

**Affiliations:** 1 Systemomics Center, College of Pharmacy, and Genomics Research Center (State-Province Key Laboratories of Biomedicine-Pharmaceutics of China), Harbin Medical University, Harbin, 150081 China; 2 HMU-UCFM Centre for Infection and Genomics, Harbin, China; 3 Department of Biochemistry and Molecular Biology, University of Calgary, T2N1N4, Calgary, Canada; 4 Department of Microbiology, Immunology and Infectious Diseases, University of Calgary, T2N1N4, Calgary, Canada; 5 Current address: Department of Biomedical Sciences, University of Calgary, T2N1N4, Calgary, Canada

**Keywords:** Ovarian cancer, Enterodiol, Enterolactone, Anti-cancer

## Abstract

**Background:**

Ovarian cancer is one of the three leading gynecological malignancies, characterized by insidious growth, highly frequent metastasis, and quick development of drug resistance. As a result, this disease has low 5-year survival rates. Estrogen receptor inhibitors were commonly used for the treatment, but only 7% to 18% of patients respond to anti-estrogen therapies. Therefore, more effective therapies to inhibit estrogen-related tumors are urgently needed. Recently, phytoestrogens, such as lignans with estrogen-like biological activities, have attracted attention for their potential effects in the prevention or treatment of estrogen-related diseases. Enterodiol (END) and enterolactone (ENL) are mammalian lignans, which can reduce the risk of various cancers. However, the effects of END and ENL on ovarian cancer are not adequately documented.

**Methods:**

We used in vitro assays on the ES-2 cell line to evaluate the inhibiting effects of END and ENL on ovarian cancer cell proliferation, invasion and migration ability and in vivo xenograft experiments on nude mice to validate the anticancer effects of END and ENL.

**Results:**

The in vitro assays demonstrated that high-dose END and ENL could obviously inhibit ovarian malignant properties, including cancerous proliferation, invasion, and metastasis. Compared to END, ENL behaved in a better time-dose dependent manner on the cancer cells. The in vivo experiments showed that END (1 mg/kg), ENL (1 mg/kg) and ENL (0.1 mg/kg) suppressed tumor markedly, and there were statistically significant differences between the experimental and control groups in tumor weight and volume. Compared to END, which have serious side effects to the animals at high concentration such as 1 mg/kg, ENL had higher anticancer activities and less side effects in the animals than END at the same concentrations, so it would be a better candidate for drug development.

**Conclusion:**

END and ENL both have potent inhibitory effects on ovarian cancer but ENL possesses a more effective anti-cancer capability and less side effects than END. Findings in this work provide novel insights into ovarian cancer therapeutics with phytoestrogens and encourage their clinical applications.

## Background

Ovarian cancer is the fifth leading cause of cancer among females but ranks first in mortality among all gynecological cancers [1]. In the United States, 22,820 new ovarian cancer cases with 14,240 deaths were reported in 2016 [2]. The five most common types of this disease are high-grade and low-grade serous, endometrioid, mucinous, and clear cell carcinoma. All ovarian cancers have an insidious onset with hardly noticeable progresses until advanced stages (Stages III and IV). As a result, at the time when the disease is finally diagnosed, peritoneal metastasis often has already occurred. The standard treatment comprises of surgery to remove all macroscopic tumors and systemic chemotherapy to clear or suppress remaining cancer cells [3]. Generally, ovarian cancers are sensitive to platinum agents, and taxane/platinum combined regimens are often used in first-line chemotherapy, although resistance to platinum reagents is common at advanced stages. Furthermore, conventional chemotherapy is usually cytotoxic with a myriad of side effects. Therefore, more effective and less cytotoxic therapies to treat ovarian cancers are urgently required [4].

Phytoestrogens, including mainly isoflavones and lignans, are abundant in food materials and are considered to have preventive and therapeutic effects against various cancers [5, 6]. Enterodiol (END) and enterolactone (ENL) are extensively investigated lignans for their potential medical uses [7, 8]. We and other authors have reported the production of END and ENL by human intestinal microbiota through biotransformation from flaxseeds (seeds of *Linum usitatissimum* L.) [9–12]. END and ENL both can reduce the risk of hormone-dependent cancers in the breast [9, 13], uterus [14], and prostate [15]. The anticancer activities of flaxseed lignans have been attributed to two mechanisms, i.e., antioxidant and hormone receptor modulating effects [16, 17]. END and ENL act as antioxidants against DNA damage and lipid peroxidation in cancer and probably also contribute to the reduction of hypercholesterolemia, hyperglycemia, and atherosclerosis [18]. Of specific significance, END and ENL can mimic the structure of human estrogens to upregulate or downregulate the functions of estrogen receptors (ERs) [19]. At relatively low doses, END and ENL exhibit the estrogenic activity, while at higher doses they appear to be antiestrogenic. The “biphasic effects” might be caused by protein kinase inhibitors at low doses and the topoisomerase activity at higher doses respectively [7, 20].

There is a considerable body of evidence from epidemiological studies correlating high concentrations of lignans in body fluids with a low incidence of hormone-dependent tumors, in particular breast cancer [21, 22]. For example, a follow-up study of postmenopausal breast cancer patients showed that postmenopausal breast cancer patients having high enterolignan levels may have a better survival [23]. In another study on serum concentrations in correlation with dietary intake of flaxseed, postmenopausal women consuming flaxseeds had decreased serum 17β-estradiol and estrone sulfate concentrations and lowered breast cancer risks [24]. Additionally, numerous in vitro studies and in vivo animal experiments demonstrated potent anticancer effects of END and ENL, such as work on breast cancer cell lines MCF-7 and MDA MB 231, which demonstrated the anti-metastatic activity of ENL, probably by inhibiting cell adhesion, cell invasion and cell motility through down-regulating MMP-2, MMP-9 and MMP-14 gene expression [25]. Researchers measured the urinary ENL level in postmenopausal women as well as in breast cancer patients, who were treated with breast cancer removal surgery, and found that breast cancer patients had significantly lower ENL levels compared to the control group, suggesting that ENL might be involved in reducing the risk of breast cancer [26]. In another study, flaxseed, which is a good source of END and ENL, administered in a basal high-fat diet reduced the nuclear aberration and epithelial proliferation in female rat mammary gland, suggesting a protective effect of flaxseed against breast cancer [27]. Similar results have been found in colon cancer, in which lignans caused cell proliferation inhibition and induced apoptosis [28].

Nude mouse models have been used to evaluate the therapeutic effects of END, ENL and other phytoestrogens. A study based on a model of human breast cancers in nude mice showed that cancer animals treated with tamoxifen and fed with flaxseeds or ENL exhibited decreased IL-1β levels compared to controls, which would suppress tumor angiogenesis and reduce microvessel density in vivo [29]. Another breast cancer mouse model using MCF-7 cells showed that ENL had potent effects against breast cancer growth, whereas GEN as the control did not [30]. Additionally, compared to genistein, END and ENL are more suitable for prolonged treatment [9]. The effect of ENL on colon cancer growth and its mechanisms of action was investigated by detecting apoptosis- and proliferation-related proteins and establishing colon cancer mouse models [31]. ENL at a dose of 10 mg/kg could suppress human colon cancer cell growth both in vitro and in vivo [31].

Numerous findings have been reported on END and ENL with different types of tissues or cancers, such as those on lignans with hen ovaries [32, 33], but work about the effects of END and ENL on human ovarian cancers is lacking. In this study, we investigated the possible anti-cancer potential of END and ENL by in vivo and in vitro experiments. Our results demonstrated excellent anticancer effects of both ENL and END, although ENL exhibited higher efficacy and less side-effect than END in ovarian cancer treatments.

## Methods

### Cell culture

The ES-2 cell line was purchased from Wuhan Procell life Science & Technology Co Ltd. and cultured in McCoy’s 5A medium supplemented with 10% FBS, penicillin-streptomycin (100 units/ml and 100 μg/ml, respectively) at 37 °C in 5% CO_2_. Culture medium was changed every other day and cells were passaged at 3–4 day intervals when they reached confluence.

### MTT assay

ES-2 cells were seeded in 96 well plates at a density of 10,000 per well in 180 μl medium. After 24 h incubation, the cells were treated with END or ENL at concentrations ranging from 10^−3^ to 10^−6^ mol/L for 24, 48 or 72 h and cell proliferation indices were measured by 3-(4,5-dimethyl thiazol-2-yl)-2,5-diphenyl tetrazolium bromide (MTT) assay. After each incubation period, 20 μL of MTT (5 mg/mL in PBS) was added to each well and the plates were incubated for further 4 h at 37 °C. Media were discarded and 150 μl of dimethyl sulfoxide (DMSO) was added. The absorbance was measured at 492 nm.

### Trypan blue assay

Trypan blue exclusion assay was performed to assess the effects of END and ENL on the viability of malignant cells. After 48 h treatment by different concentrations of END or ENL (10^−3^, 10^−4^, 10^−5^, or 10^−6^ mol/L), cells seeded in the 24-well plate were trypsinized, then the cell suspension and trypan blue were 1:1 mixed and viable cells were counted by a hemocytometer(Countstar,Ruiyu Biotech Co., Ltd.,Shanghai). Each assay was performed in triplicate.

### Transwell assay

The effects of END or ENL on the invasive properties of ES-2 cells were evaluated by transwell assays, with the transwell chambers consisting of 8 mm membrane filters (8 μm; Corning Costar, Cambridge, MA) in 24-well plates. A total of 40,000 cells in serum-starved overnight culture were suspended with END or ENL at concentrations of 10^−3^ mol/L, 10^−4^ mol/L, 10^−5^ mol/L and 10^−6^ mol/L diluted by serum-free medium, and were added to the upper compartment with a matrigel-coated (1 mg/mL) polycarbonate membrane, and a volume of 0.5 ml of 10% FBS-containing medium was added to the lower chamber as a chemoattractant. After 48 h of incubation, cells on the upper surface of the filters were carefully wiped off with a cotton swab, and others that had migrated to the bottom of the membrane were fixed with 90% methanol and stained with 0.1% crystal violet. Stained cells were counted under a microscope in a blinded manner at 5 randomly selected areas (20×). Experiments were done in triplicates.

### Wound healing assay

The wound healing assay was performed to determine the cell migration ability. One day before scratching, cells were seeded in 24-well plates. When the monolayer reached confluency, a straight wound was then artificially created on the monolayer with a sterile 10 μl pipette tip. After that, cells were gently washed two times with phosphate-buffered saline (PBS), treated with 10^−3^ mol/L, 10^−4^ mol/L, 10^−5^ mol/L or 10^−6^ mol/L END or ENL diluted by non-serum fresh medium and were cultured for 48 h. At least five random nonoverlapping images of the wounded areas were taken at 0 and 48 h, captured by inverted microscope (100×) and quantitated using Image J software (National Institutes of Health, Bethesda, MD).

### Animal housing conditions and study protocol

BALB/c nude mice were raised on purified air laminar flow shelves in the SPF laboratory, with constant temperature (25 ± 2)°C and humidity (45% to 50%). The study has been reviewed by Harbin Medical University Ethics Committee and strictly abided by Directive 2010/63/EU in Europe on the protection of animals used for scientific purposes. A total of 35 female mice, weighing 16 ± 18 g, 4–8 weeks old, were randomly divided into the control, END (1 mg/kg, 0.1 mg/kg) and ENL (1 mg/kg, 0.1 mg/kg) groups, 7 in each group. Cultured cancer cells at the logarithmic growth phase were trypsinized, collected by centrifugation at 1000 r/min for 5 min, re-suspended in the serum-free medium, counted for the number of living cells, and diluted for a cell concentration of 1 × 10^6^ cells/ml. Each mouse was injected with 50 ul cell-suspension at the right hind leg.

Animal activities such as eating and defecation were observed daily and body weight was recorded once in 2 days after cancer cell inoculation. When the tumor began to be visible, we measured the longest diameter (a) and the shortest diameter (b) of the tumor nodes with vernier caliper and recorded the data in detail. Mice in experimental groups were injected with END or ENL at 1 mg/kg or 0.1 mg/kg separately, once per 2 days by intra-tumor injection method. At the same time, mice in control group were injected with PBS (1ul/g), once per 2 days. On day 32, all mice were sacrificed and the tumor volume, tumor weight, body weight and spleen weight were measured. We dissected the mice to check tissue invasion around the tumor and the metastasis of superficial lymph node and viscera.

### Statistical analysis

Student’s *t*-test, Chi square test and One-ANOVA were performed to determine statistically significant differences in MTT, Wound Healing, Trypan Blue, Transwell assays and in vitro experiments across different END and ENL concentrations as appropriate. SPSS statistical software version 17.0 and GraphPad Prism software were used in this work. A *P*-value of <0.05 was considered statistically significant. Our study closely followed the line of randomness and preciseness to ensure reproducibility.

## Results

### END and ENL both inhibited ES-2 cell growth: ENL was more effective

**Fig. 1:**
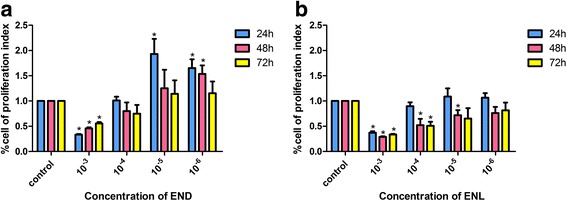
Growth inhibition of ES-2 cells by END and ENL revealed by MTT assays. **a** Cell proliferation index determined after END treatment for 12, 24 or 48 h. **b** Cell proliferation index determined after ENL treatment for 12, 24 or 48 h. Relative cell numbers were normalized to the control. Results were obtained from three independent experiments. *P < 0.05, compared with the control

END and ENL both inhibited the growth of ES-2 cells at 10^−3^ mol/L; at lower concentrations (up to 10^−5^ mol/L), ENL but not END remained active. Compared with END, ENL exhibited a dose- and time-dependent manner in its activities as judged by cell proliferation percentages in comparison with the control (Fig. 1).

### END and ENL inhibited ES-2 cell viability at a high concentration

**Fig. 2:**
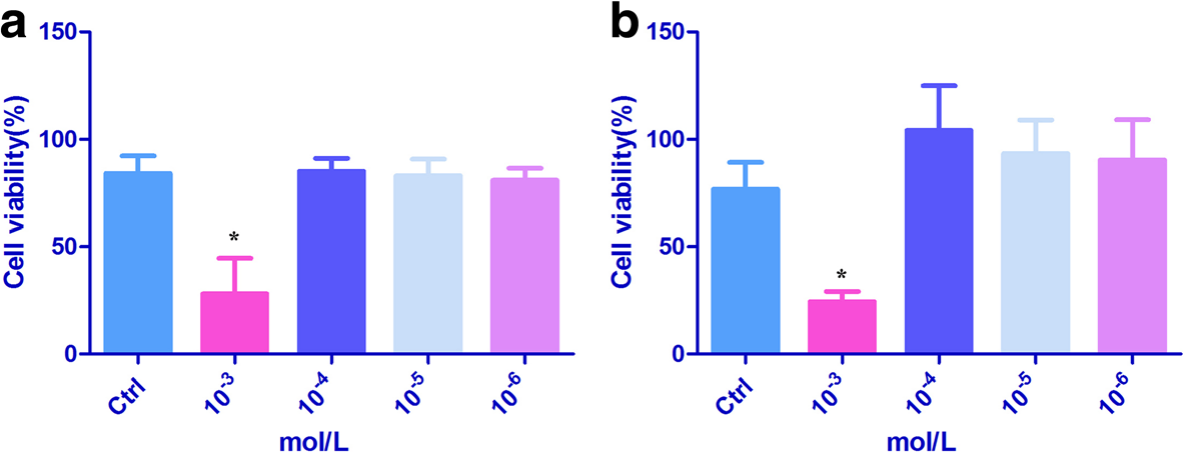
Viability inhibition of ES-2 cells by END and ENL revealed by Trypan *blue* assays. **a** Cell viability (%) after END treatment. **b** Cell viability (%) after ENL treatment. Results were obtained from three independent experiments. *P < 0.05, compared with the control

Trypan blue stain assay was used to determine whether END or ENL could affect ES-2 cell livability and revealed that END and ENL both could inhibit cell viability remarkably, 28.023% and 24.38%, respectively, at the concentration of 10^−3^ mol/L; no obvious changes were observed at lower concentrations (Fig. 2).

### Effects of END and ENL on the migration of ES-2 cells

**Fig. 3:**
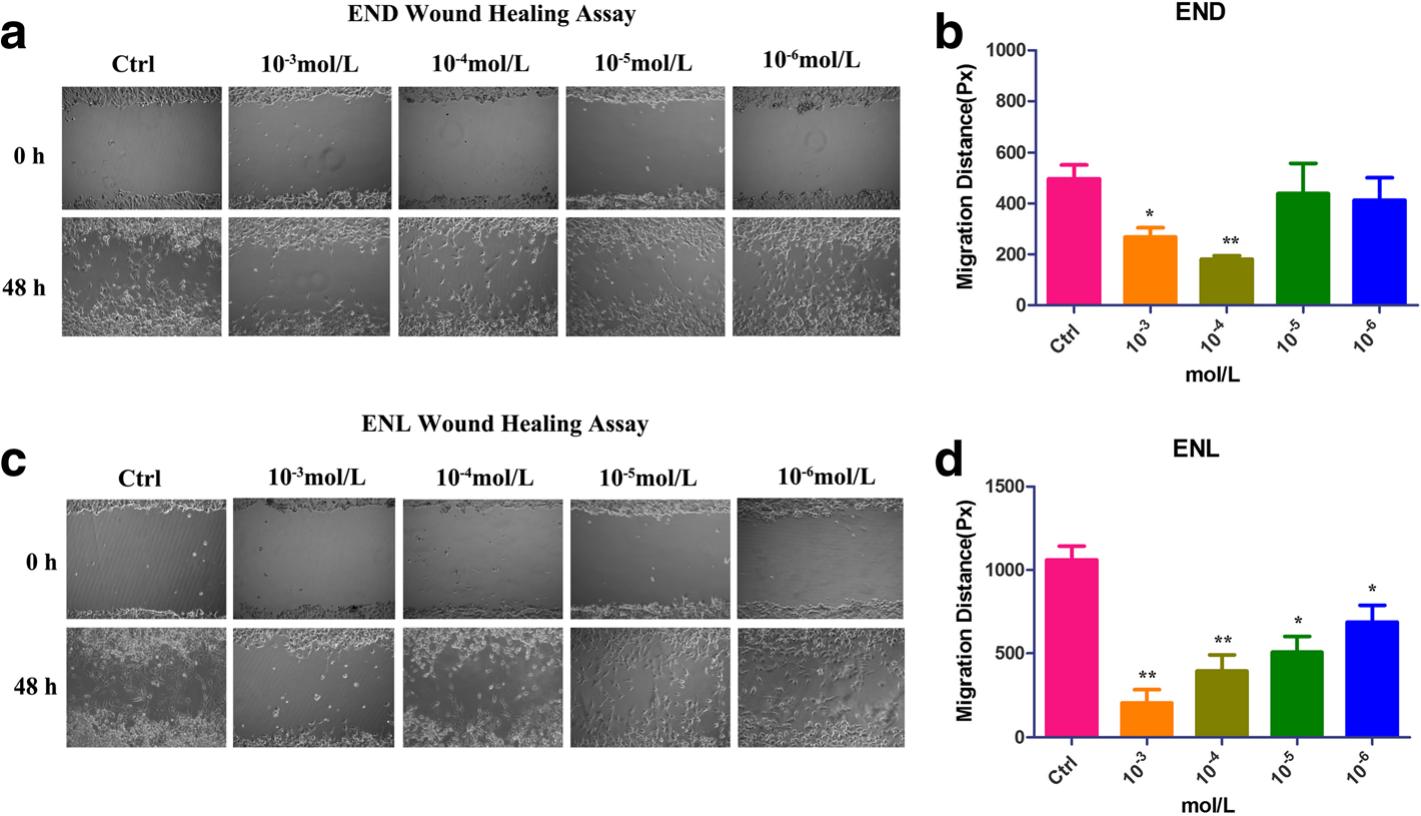
Migration inhibition of ES-2 cells by END and ENL quantified by Wound Healing assay. Cells were scratched and treated with END (**a**) and ENL (**c**). Photomicrographs were taken at 0 h and 48 h after scratching. **b**, **d** Quantitation of wound healing assay by END (b) and ENL (**d**). Results were obtained from three separate experiments. *P < 0.05, compared with the control

Since cell migration is an initial step in the metastatic process of ovarian cancer, the effects of END and ENL against the migration of ES-2 cells were examined by wound healing assay. We found that treatment with END or ENL both inhibited the migration of the cells at 10^−5^ mol/L or higher concentrations compared to the control group (Fig. 3a, b). Consistently, Wound healing assay results also showed that the migration distances appeared to be dose-dependent in the presence of ENL (10^−3^ mol/L-10^−4^ mol/L, *p* < 0.01; 10^−5^–10^−6^ mol/L, *p* < 0.05) (Fig. 3c, d). On the other hand, treatment with END decreased the migration distance significantly at the concentrations of 10^−3^ mol/L and 10^−4^ mol/L, but not at 10^−5^ mol/L or 10^−6^ mol/L (Fig. 3a, b). Although cells were cultured by FBS-free medium while different treatments, both proliferation and migration might be contributed to scratches healing as shown in Fig. 3a, c.

### Effects of END and ENL on the invasiveness of ES-2 cells

**Fig. 4:**
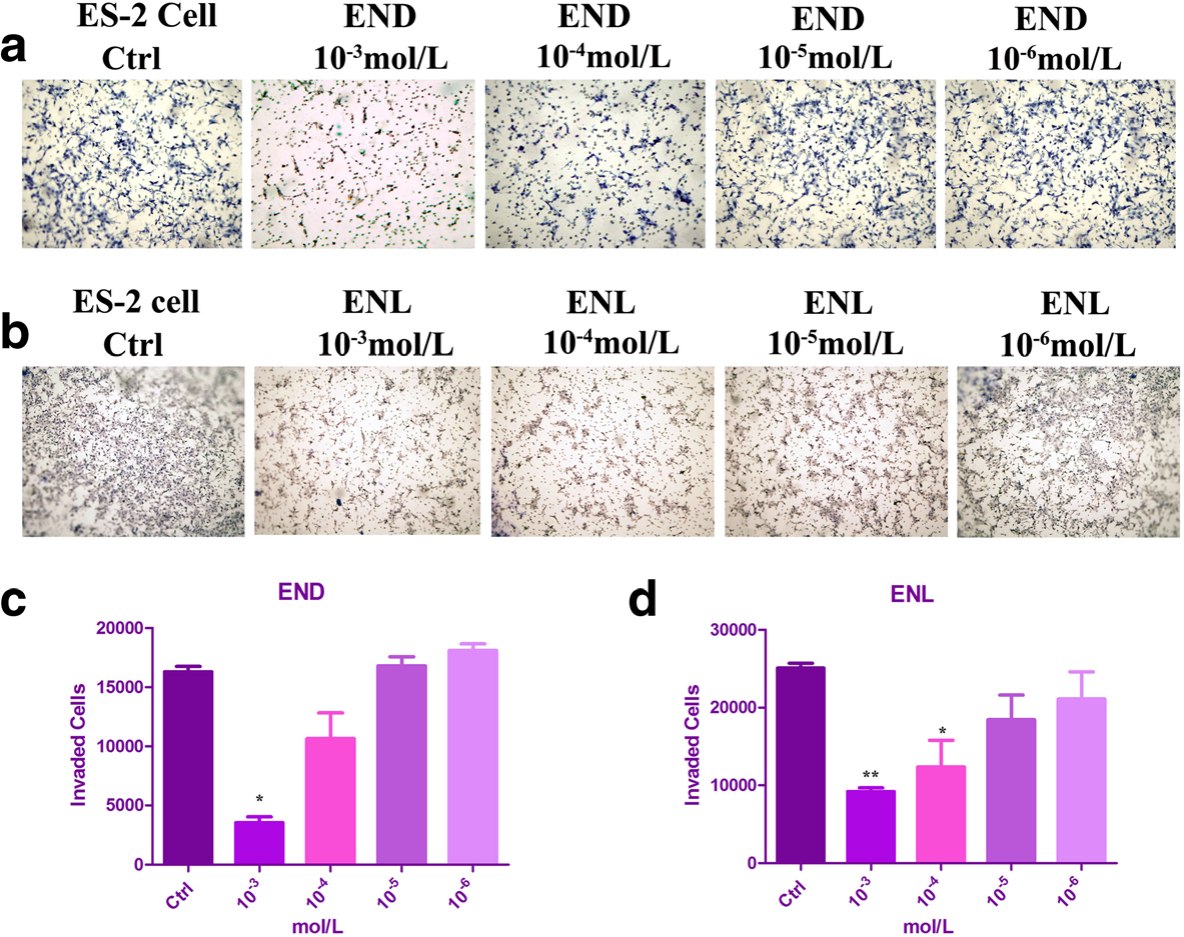
Invasiveness of ES-2 cells quantified by Transwell. Cells were plated in the upper chamber with END (**a**) and ENL (**b**). Pictures were taken under the microscopy after 48 h of treatments. The invading cells treated by END (**c**) and ENL (**d**) were quantitated. Results were obtained from three separate experiments. *P < 0.05, compared with control

Cancer cell migration may lead to invasion of normal tissues. To determine whether END and ENL could also inhibit the invasive potential of ES-2 cells, transwell matrigel invasion assay was performed. By this assay, ES-2 cells that invaded through the matrigel were counted. Treatment with different concentrations of END and ENL inhibited the invading cells by a dose-depend manner compared with control. As shown in the transwell assay, END at 10^−3^ mol/L and ENL at 10^−3^ mol/L or 10^−4^ mol/L all decreased cell invasion significantly compared to the control groups. However, surprisingly, END at 10^−5^ mol/L and 10^−6^ mol/L stimulated and enhanced the invasion (Fig. 4).

### Effects of END and ENL on tumor growth in the nude mice and on general health of the animals

**Fig. 5:**
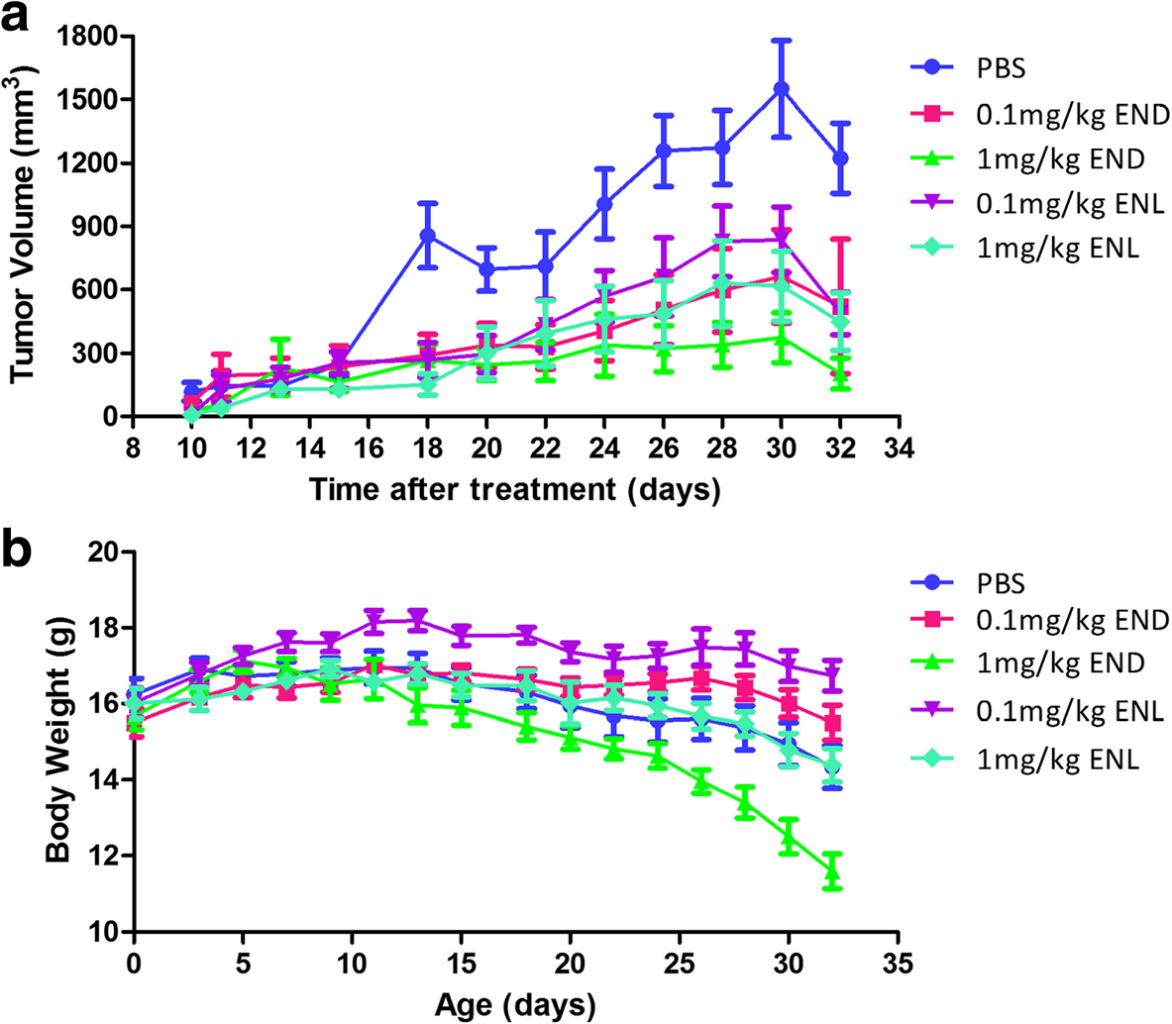
Tumor growth and body weight changes during END and ENL treatment. **a** The tumor volume curve starting to be measured on day 10 after cancer cell inoculation. The tumor size was calculated according to the formula V = 0.5 ab^2^ (see details in the main text). **b** The body weight curve, which shows that 1 mg/kg END group dropped drastically, while other groups were relatively stable during the 32 days. Both body weight and tumor volume were measured once every two days

We used the human ES-2 ovarian cancer cells to generate solid tumors in the nude mice. The rate of xenograft was 100%, with subcutaneous tumors becoming visible on day 7 after injection of the cancer cells. On day 10 after injection, we began the treatment with END or ENL at 0.1 mg/kg or 1 mg/kg, administered once every other day until day 32, with PBS as the control. We observed a significant reduction in the tumor volume in the experimental groups treated with END or ENL compared to the PBS group (Fig. 5a). Body weights of the animals were also measured every alternate day from day 1 to day 32, which declined in all groups but with different patterns among the groups (Fig. 5b). As what was weighted is in fact the sum of the tumor and the rest of the body, the interpretation of weight changes should be made very carefully. For example, in the group of mice treated with 1 mg/kg END, the body weight fell down the most remarkably as shown in Fig. 5b, which could be interpreted to be the combined consequence of suppressed tumor growth due to the anticancer activities of END and of weight loss of the remaining part of the body due to the side-effects of END. As a result, the overall body weight changes should be evaluated by considering both aspects of the effects of the lignans, which explains the overlapping phenomenon of the body weight curves between mice receiving 1 mg/kg ENL and the controls receiving PBS (Fig. 5).

### ENL as a more suitable anti-cancer agent than END in vivo

**Fig. 6:**
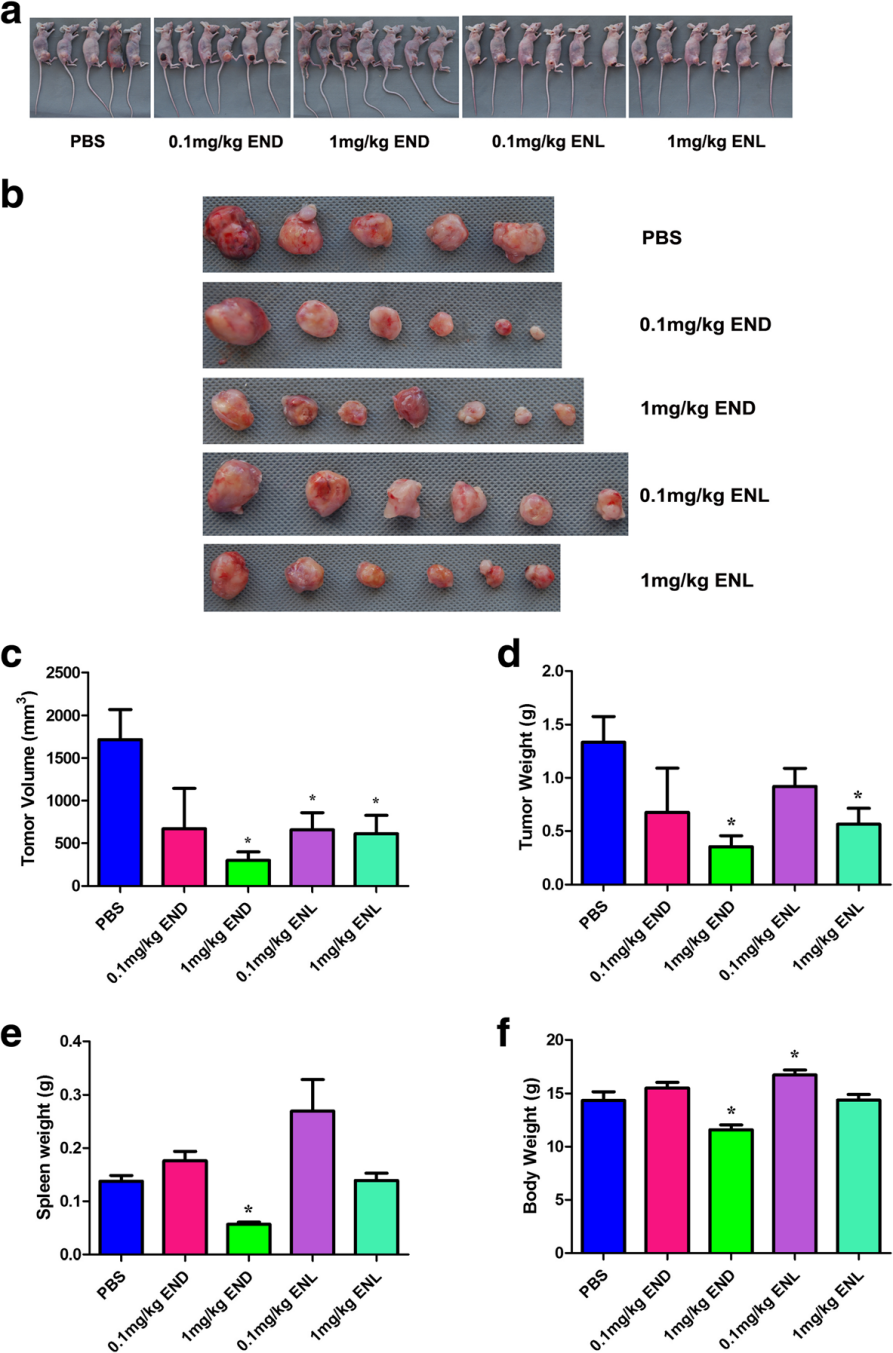
Comparisons of the final body weight, spleen weight, tumor weight and tumor volume of the animals treated with END or ENL at necroscopy. The sacrificed animals (**a**) and tumors (**b**), and statistics of tumor size (**c**), tumor weight (**d**), spleen weight (**e**) and body weight (**f**)

At the end of experiment, all mice were sacrificed for histological examinations of the tumor tissues (Fig. 6a, b). By treatments with END and ENL, the tumor volumes were reduced compared to the PBS control group, consistent with the curves (see Fig. 5). The final tumor volume, tumor weight, body weight and spleen weight were measured and recorded upon necroscopy on day 32 (Fig. 6c-f), showing that the mean tumor volume and tumor weight were both significantly reduced in the 1 mg/kg END and 1 mg/kg ENL-treated groups compared to the untreated and low-concentration groups. However, since 1 mg/kg END caused a marked drop in body weight (Mean ± SEM: 11.59 ± 0.4554 *N* = 7; the weight loss was most pronounced in the spleen as shown in Fig. 6e) compared with PBS group (Mean ± SEM: 0.1378 ± 0.01069 *N* = 5) but 1 mg/kg ENL did not (Mean ± SEM: 0.1390 ± 0.01391 *N* = 6), it is evident that ENL as an anti-cancer agent would be more suitable for medical use than END.

## Discussion

Ovarian cancer is the most lethal gynecologic malignancy among women, with metastasis and drug resistance being the major causes of disease exacerbation [34, 35], which calls for novel therapeutic modalities. Lignans with anticancer activities, abundant in food materials such as those in flaxseeds [11, 12], have great potential for cancer prevention and treatment as they may reduce the incidence of various cancer types [14, 31, 36, 37]. However, their specific effects on ovarian cancer have not been adequately documented. In this study, we characterized the anti-cancer effects of END and ENL on ovarian cancer both in vitro and in vivo.

Epidemiologic studies have suggested that phytoestrogen consumption may decrease ovarian cancer risk [38], although very little has been documented specifically about END or ENL on human ovarian cancer by molecular methods. The available reports on flaxseed lignans focus mostly on animal normal ovaries, not cancer, such as the work on END and ENL inhibiting estrogen metabolism by down-regulating IGF, AKT and NF-kB pathway while up-regulating CYP1A1 expression to protect hen ovary [22] and the work on flaxseed feeding to inhibit inflammation as well as the estrogen related pathway and block inflammation along with the microenvironment changes in the hen ovaries [23].

We showed in this study that END and ENL could inhibit ES-2 cell proliferation, viability, migration and invasiveness at a high concentration (10^−3^ mol/L), although ENL but not END suppressed the ES-2 cells in a dose- and time-dependent manner as evaluated by MTT, wound healing and Transwell assays, which is consistent with previously reported studies in other cancer types [7, 9, 39]. Many studies have mentioned the estrogenic and antiestrogenic activity of phytoestrogens, which inferred that at higher doses, the phytoestrogens might serve as protein kinase inhibitors or show topoisomerase activity [7]. According to previous studies, we could further explain our work that END performed an estrogenic activity at lower concentrations (10^−4^ mol/L, 10^−5^ mol/L, 10^−6^ mol/L), while higher concentration (10^−3^ mol/L) inhibited cell proliferation significantly. In contrast, ENL served as a time- or does- dependent inhibitory mode in most in-vitro experiments.

The in vivo assay in this study revealed a quite remarkable fact: whereas END at 1 mg/kg and ENL at both 1 mg/kg and 0.1 mg/kg could remarkably suppress tumor growth, END (1 mg/kg) may bring about serious side effects as evaluated by weight loss of both the whole body and the spleen, suggesting that ENL but not END could be a potential therapeutic agent against ovarian cancer for its better effectiveness and lesser side effects.

## Conclusion

END and ENL both have potent inhibitory effects on ovarian cancer, as evidenced by in vitro and in vivo investigations in this study, but ENL possessed a more effective anticancer capability and less side effects than END. Findings in this work provide novel insights into ovarian cancer therapeutics with phytoestrogens and encourage their clinical applications.

## Abbreviations

AKT: Protein kinase B
ANOVA: Analysis of Variance
CYP1A1: Cytochrome P450, family 1, subfamily A
DMSO: Dimethylsulphoxide
END: Enterodiol
ENL: Enterolactone
ERs: Estrogen receptors
FBS: Fetal Bovine Serum
GEN: Genistein
IGF: Insulin-like Growth Factor
IL-1β: Interleukine-1β
MMP14: Matrix metalloproteinase-14
MMP2: Matrix metalloproteinase-2
MMP9: Matrix metalloproteinase-9
NF-kB: Nuclear factor kappa B
PBS: Phosphate-buffered Saline
SEM: Standard Error of the mean
SPF: Specific Pathogen-Free
SPSS: Statistical Product and Service Solutions
